# WHO/INRUD prescribing indicators and prescribing trends of antibiotics in the Accident and Emergency Department of Bahawal Victoria Hospital, Pakistan

**DOI:** 10.1186/s40064-016-3615-1

**Published:** 2016-11-08

**Authors:** Muhammad Atif, Muhammad Azeem, Muhammad Rehan Sarwar, Samia Shahid, Sidra Javaid, Huria Ikram, Uzma Baig, Shane Scahill

**Affiliations:** 1 Department of Pharmacy, The Islamia University of Bahawalpur, Bahawalpur, Punjab Pakistan; 2 School of Management, Massey University, Auckland, New Zealand

**Keywords:** Rational, Antimicrobial use, Antimicrobial resistance, Prescriptions, Irrational prescribing

## Abstract

A descriptive, retrospective and cross sectional study was conducted to assess the prescribing practices and antibiotic use patterns in the Accident and Emergency department of the Bahawal Victoria Hospital, Bahawalpur, Pakistan. A sample of 4320 prescriptions (systematic random sampling) was drawn out of a total of 1,080,000 prescriptions written during the period 1st January–31st December 2014. The standard World Health Organization/International Network for Rational Use of Drugs prescribing indicators were used to determine the prescribing practices of physicians. Published ideal standards for each of the indicators were used to identify irrational drug use. We also utilized an additional indicator to report the percentage share of antibiotics prescribed. The average number of drugs prescribed per encounter was 2.3 (SD = 1.3) (optimal value 1.6–1.8). Drugs prescribed by generic name occurred 83.1% of the time (optimal value 100%). Antibiotics and injections were prescribed 52.4% (optimal value 20.0–26.8%) and 98.0% (optimal value 13.4–24.1%) of the time respectively. Drugs prescribed from the Essential Drugs List equated to 81.5% (optimal value 100%). Out of 52.4% (n = 2262) prescriptions with antibiotics prescribed, 77.7% (n = 1758) had one antibiotic, 22.1% (n = 499) included two antibiotics, and 0.2% (n = 5) had three antibiotics. Cephalosporins were the most commonly prescribed class of antibiotics (81.5%) followed by penicillins (6.4%) and fluoroquinolones (6.2%). Among the individual antibiotics, ceftriaxone contributed the highest percentage share at 71.8% followed by cefotaxime (5.6%) and metronidazole (4.7%). The most frequently prescribed antibiotic combination was ciprofloxacin with metronidazole (52.1%). Irrational prescribing practices were common. Continuous education and training of physicians is required to ensure rational prescribing at Bahawal Victoria Hospital in the future.

## Background

The importance of assessment and evaluation of quality in healthcare is gaining global recognition and medicines play a pivotal role in the healthcare delivery system. The appropriate use of medicines is a crucial element in the quality of health outcomes and in the appropriate medical care provided to patients (World Health Organization 1993). The most common causes of irrational medicine use are; self-medication, polypharmacy, inappropriate use of antibiotics, overuse of injectable medicines and the prescribing of medicines without following relevant clinical practice guidelines (World Health Organization 2002). Furthermore, there are numerous factors that influence irrational prescribing including; patients, healthcare professionals (HCPs), the working environment, the drug supply system (including industrial impacts), legal regulations, information and misinformation about medicines, and profiteering intentions by selling more medicines (Geest et al. 1991; Spurling et al. 2010).

 The fundamental step to limiting the irrational use of medicines is to quantify the extent to which this is occurring. It is particularly important with antibiotics as resistance continues to climb and the armamentarium of new antibiotics coming to market is not on the increase. In the 1990s, the World Health Organization (WHO) in collaboration with the International Network of Rational Use of Drugs (INRUD) developed a set of indicators to measure the performance of healthcare facilities related to the utilization of drugs (World Health Organization 1993). In developing countries, assessment of drug use patterns through the WHO/INRUD indicators is on the increase which is promising (Hogerzeil et al. 1993) with the indicators having been successfully implemented in more than 30 developing countries (Laing et al. 2001).

With regard to the rational use of antibiotics, the WHO has defined this as ‘the cost-effective use of antibiotics which maximizes clinical therapeutic effect while minimizing both drug-related toxicity and the development of antimicrobial resistance (AMR)’ (World Health Organization 2001a, b). The supervision of antimicrobial use has environmental, economic and clinical implications (World Health Organization 2012). Between 30 and 50% of all hospitalized patients are prescribed at least one antibiotic and this class constitutes more than 30% of the hospital budget (Vlahovic-Palcevski et al. 2000). According to another estimate, 20–50% of antimicrobial utilization is inappropriate (Čižman 2003) and this has a significant effect on the quality of health services provided (Shao-Kang et al. 1998), treatment expenses (Segade 2000) and frequency of adverse drug reactions (ADRs). It has also been estimated that around one quarter (25%) of total ADRs can be attributed to antimicrobial use (Beringer et al. 1998). Despite these concerns, the most alarming problem associated with irrational prescribing of antimicrobials is the development of resistance (Dellit et al. 2007). This problem is exacerbated by the limited number of new antimicrobials coming through the development pipeline of large pharmaceutical companies (O’Neill 2015).

In the United States (US), the economic burden of AMR amongst outpatients is estimated to be between $400 million and $18.6 billion. The costs associated with inpatients are thought to be much higher (Okeke et al. 2005). According to the WHO, AMR costs in Europe are estimated to be €9 billion per annum (World Health Organization 2011). Unfortunately there is a scarce literature on the fiscal burden of AMR in developing countries such as Pakistan. The expenses associated with AMR could inflict significant fiscal damage, not only for the governments but also for patients living in developing countries. Thus, monitoring and supervision of antimicrobial utilization has significant benefit to society through the saving of valuable resources (World Health Organization 2012).

The aim of this study was to assess the prescribing practices in the Accident and Emergency (A & E) department of the Bahawal Victoria Hospital (BVH), Bahawalpur, Pakistan. We also report the antibiotic use patterns in this same context. The results of this study could assist the hospital administrators to develop and implement appropriate interventions to improve rational prescribing of medicines in the A & E department of the BVH. The study findings may also be applicable to other hospitals that are suspected to have similar drug use practices.

## Methods

### Study setting

This study was carried out in the A & E department of the BVH, Bahawalpur, Pakistan. The BVH is a 1600 bed tertiary care hospital with established surgical and medical facilities. A total of 350 physicians, 10 pharmacists, 400 nurses and 3000 paramedical staff serve an average of 90,000 patients per month. The A & E department was established in 2005 with a capacity of 100 beds. The staff includes 25 doctors, one pharmacist and 54 nurses who provide care for between 2000 and 2500 patients per day.

### Study design and outcome measures

A retrospective, cross-sectional study design was employed to evaluate the prescribing practices for all medicines, as well as antibiotic utilization patterns. The optimal values for the prescribing indicators were adopted from previous studies (Desalegn 2013; Atif et al. 2016b). The prescribing indicators include; the average number of drugs prescribed per encounter (optimal value 1.6–1.8), the percentage of drugs prescribed by generic name (optimal value 100%), the percentage of encounters where an antibiotic was prescribed (optimal value 20.0–26.8%), the percentage of encounters where an injection was the route of administration (optimal value 13.4–24.1%), and the percentage of drugs prescribed from the Essential Drugs List (EDL) or some other recognized formulary (for which the optimal value is 100%). Another indicator was used to determine antibiotic prescribing patterns at the A & E department of the hospital.

*Inclusion* All prescriptions written for the patients attending the A & E department of the BVH from 1st January 2014–31st December 2014 irrespective of age, gender and diagnosis were included in the study.

### Sample size and data collection method

The sample size for this study was 4320 prescriptions collected retrospectively from 1,080,000 written during the 1-year period of study. The prescriptions written each month were separated and then 360 prescriptions from each month were selected using a systematic random sampling technique.

The data relating to the prescribing indicators was noted for each prescription and then recorded into a standard WHO prescribing indicator form (World Health Organization 1993). A data collection form was developed to capture antibiotic utilization patterns and data was recorded into it. The Anatomical Therapeutic Chemical (ATC) classification system was used for the classification of antibiotic (WHO Collaborating Centre for Drug Statistics Methodology 2014). The data was collected by four trained research associates during the months of January–March 2015.

### Statistical analysis

Statistical Package for Social Sciences (IBM SPSS Statistics V21.0) was used for the analysis of data. Descriptive statistics such as frequencies, percentages, averages and standard deviations (SD) were used to present data.

## Results

### Prescribing indicators

The average number of drugs per encounter was 2.3 (SD = 1.3). The percentage of drugs prescribed by generic name was 83.1% (SD = 1.1). The percentage of encounters with an antibiotic prescribed was 52.4% (SD = 0.5) and 98% (SD = 0.3) of drugs were given by the injectable route. Over three quarters (81.5%) of drugs were prescribed from the EDL or some other formulary (SD = 1.1) (Table 1).

**Table 1:** WHO/INRUD prescribing indicators in the Accidents and Emergency department of the Bahawal Victoria Hospital (n = 4320)

Indicator	Total	Value (SD)	Optimal level
The average number of drugs per encounter	10,075	2.3 (1.3)	1.6–1.8
% Drugs prescribed by generic name	8368	83.1% (1.1)	100%
% Encounters with an antibiotic	2262	52.4% (0.5)	20.0–26.8%
% Encounters with an injection	4216	98% (0.3)	13.4–24.1%
% Drugs from essential drugs list	8208	81.5% (1.1)	100%

### Patterns of antibiotic prescribing

Over half of prescriptions included antibiotics (52.4%, n = 2262) and of these over three-quarters (77.7%, n = 1758) had one antibiotic, 22.1% (n = 499) included two antibiotics and 0.2% (n = 5) had three antibiotics. Cephalosporins (81.5%), penicillins (6.4%) and fluoroquinolones (6.2%) were the most frequently prescribed antibiotic classes (Table 2).

**Table 2:** Most commonly prescribed antibiotic classes

Class of antibiotics	ATC code	Frequency (%)
Cephalosporins	J01D	1425 (81.5)
Penicillins	J01C	112 (6.4)
Fluoroquinolones	J01M	109 (6.2)
Others	J01X	94 (5.4)
Aminoglycosides	J01G	7 (0.4)
Macrolides	J01FA	1 (0.1)
Total	–	1748 (100)

*ATC* Anatomical Therapeutic Chemical classification system

Ceftriaxone was the most commonly prescribed (71.8%) antibiotic. Thereafter, cefotaxime (5.6%), metronidazole (4.7%) and amoxicillin (4.7%) were most common (Table 3).

**Table 3:** Most commonly prescribed antibiotics

Antibiotic	ATC code	Frequency (%)
Ceftriaxone	J01DD04	1254 (71.8)
Cefotaxime	J01DD01	97 (5.6)
Amoxicillin and enzyme inhibitor	J01CR02	82 (4.7)
Metronidazole	J01XD01	82 (4.7)
Ciprofloxacin	J01MA02	74 (4.2)
Moxifloxacin	J01MA14	34 (1.9)
Cefradine	J01DB09	26 (1.4)
Ampicillin	J01CA01	24 (1.4)
Imipenem and enzyme inhibitor	J01DH51	19 (1.1)
Cefixime	J01DD08	16 (0.9)
Vancomycin	J01XA01	12 (0.7)
Cefepime	J01DE01	11 (0.6)
Gentamicin	J01GB03	7 (0.4)
Combinations of penicillin	J01CR50	4 (0.2)
Cefazolin	J01DB04	3 (0.2)
Benzyl penicillin	J01CE01	1 (0.1)
Clarithromycin	J01FA09	1 (0.1)
Total	–	1747 (100)

*ATC* Anatomical Therapeutic Chemical classification system

The most frequently prescribed antibiotic combinations were ciprofloxacin with metronidazole (52.1%) and ceftriaxone with metronidazole (38.8%) (Table 4).

**Table 4:** Most commonly prescribed antibiotic combinations

Antibiotic combinations	Frequency (%)
Ciprofloxacin + metronidazole	236 (52.1)
Ceftriaxone + metronidazole	176 (38.8)
Amoxicillin and enzyme inhibitor + metronidazole	15 (3.3)
Ceftriaxone + amoxicillin and enzyme inhibitor	13 (2.9)
Ceftriaxone + ampicillin	13 (2.9)
Total	453 (100)

## Discussion

Irrational prescribing practices exist all over the world and eventually they lead to unwanted effects in patients (Akl et al. 2014). In this study, WHO/INRUD prescribing indicators were used to determine current prescribing practices and antibiotic use patterns in a tertiary care hospital in Pakistan. The findings from this study are important to the health system of Pakistan because they help to assess whether the BVH is following a set norm of practices to ensure optimal medication use. Only a limited number of studies are available from Pakistan on this topic and therefore our findings provide a source of baseline information for continuous monitoring of drug therapy and process improvement at the institutional level. A & E departments are often the first point of contact with the healthcare system and in Pakistan there is a considerable throughput of patients in these departments. Turnover of patients in this setting is higher than general wards and one should not underestimate the importance of the appropriate prescribing of antibiotics to ensure current levels of resistance do not continue to climb. In addition to the contribution made in the Pakistani context, our findings may also be relevant to other countries with similar drug use practices or with similar health care systems. This study may provide the impetus for academics, clinicians and hospital administrators in those countries to begin to assess the status of their own nations prescribing within A & E departments; especially as it relates to antibiotics.

### Prescribing indicators

The contents of a prescription are influenced by a prescribers’ training, their attitude towards the disease being treated and the type of healthcare system within which they work. The results of the current study revealed that the average number of drugs per prescription were 2.3 (SD = 1.3) (Table 1). This value is higher than the admissible range of 1.6–1.8 drugs per encounter. In contrast to our findings, the average number of drugs prescribed was lower in Malawi (1.8) (Gelders and World Health Organization 1992) and Zimbabwe (1.3) (Hogerzeil et al. 1993). However, the studies conducted in Afghanistan (3.9) (Ahmad et al. 1995) and India (5.6) (Akhtar et al. 2012) reported a relatively higher number of drugs per prescription which could be attributed to multiple reasons. Incompetency on the part of physicians, absence of evidence-based guidelines, incentives to the prescribers, lack of continuous medical education of the prescribers and the shortage of therapeutically correct drugs provide a few reasons. Having a higher number of drugs per prescription can adversely influence treatment outcomes as patients are more likely to be non-compliant and are at greater risk of interactions and adverse events. Moreover, prescribed medicines that are not warranted lead to fiscal implications for national healthcare systems including budget blowouts (Atif et al. 2016b).

Well-founded recommendations by the WHO regarding generic prescribing provide a safety measure for patients (Atif et al. 2016a). These recommendations clearly describe what should happen and they provide accessible information and promote effective communication among healthcare providers (Akl et al. 2014). This study demonstrates that generic prescribing was at a level of 83.1% (optimal value being 100%) (Table 1). In a number of countries generic prescribing is much lower; as in Andorra (6%) (Vallano et al. 2004) and Ecuador (37%) (Hogerzeil et al. 1993) whilst higher levels have also been reported in Timor-Leste (92%) (Stanley Chindove and Martins 2012) and Ethiopia (98.7%) (Desalegn 2013).

Our results revealed that antibiotics were prescribed on over half the prescriptions (52.2%) (optimal value 20.0–26.8%) (Table 1). This would suggest that either every second person who presents at the A & E department has an infection related issue, or that there is excessive and inappropriate prescribing of antibiotics occurring in this hospital department. To compare internationally, this value was relatively lower in other developing countries such as Bangladesh (25%) (Guyon et al. 1994) and Brazil (28.8%) (Holloway and Henry 2014). In a few countries, antibiotic prescribing was higher such as in Kenya (73.4%) (Holloway and Henry 2014), Timor-Leste (70%) (Stanley Chindove and Martins 2012), and Sudan (70.4%) (Holloway and Henry 2014). Unnecessary prescribing of antibiotics is a worldwide problem that eventually leads to ADRs and frequent hospital admissions (Beringer et al. 1998).

In our study, 98% of prescriptions included at least one injectable product (optimal value 13.4–24.1%) (Table 1). This value was much higher than the studies conducted in Afghanistan (17%) (Ahmad et al. 1995) and Kuwait (9.1%) (Awad and Al-Saffar 2010). We conducted this study in the A & E department where the excessive use of injectables may be attributed to the patients’ condition such as emergency and unconscious cases where the oral route for drug administration is often not possible. Nevertheless, excessive use of injections may lead to a higher probability of blood borne diseases (World Health Organization 2002) and injections are always more expensive than the equivalent oral formulation (Akl et al. 2014).

With regard to drugs prescribed from the EDL, our findings were comparable to other studies conducted in the Lao People’s Republic (86.2%) (Holloway and Henry 2014) and Bangladesh (85%) (Guyon et al. 1994). Rational prescribing includes the optimal use of drugs selected from the EDL which are issued by the WHO. These agents are older, time tested and available at lower cost than the originator branded drugs (Akl et al. 2014).

### Antibiotic usage patterns

There is some evidence to support the notion that antibiotic consumption is much higher in developing countries as compared to developed countries (Knobler et al. 2003; Center for Disease Dynamics Economics & Policy 2015). According to one study, 35–60% of the patients were prescribed antibiotics and less than 20% of antibiotics were prescribed appropriately (World Health Organization 2001a, b).

In this study, out of the 52.4% (n = 2262) prescriptions containing antibiotics, 77.7% (n = 1758) contained one antibiotic, 22.1% (n = 499) included two antibiotics and 0.2% (n = 5) had three antibiotics. These findings can be compared with a study conducted in Jordan (Al-Niemat et al. 2014), which showed that out of 85% prescriptions with antibiotics, 88% prescriptions had one antibiotic, 11% had two and 1% had three antibiotics. Likewise in a Turkish study involving 39.4% prescriptions with antibiotics, 73.6% prescriptions had one antibiotic, 19.6% had two, 5.7% had three and 1.1% had four antibiotics (Erbay et al. 2003). Similarly, results of a study conducted in Nepal revealed that 21% prescriptions included one antibiotic, 37% prescriptions included two, 28% prescriptions included three, 10% included four and 4% of the prescriptions included five and above antibiotics (Palikhe 2008).

Our study demonstrated that the most frequently prescribed classes of antibiotics were cephalosporins (81.5%), penicillins (6.4%) and fluoroquinolones (6.2%) (Table 2). A study performed in Saudi Arabia revealed that cephalosporins (31.9%), penicillins (24.9%) and macrolides (9.7%) were the most frequently prescribed classes of antibiotics (Mohajer et al. 2011). In a similar manner, a study from Turkey reported that cephalosporins (19.9%) were the most commonly prescribed class followed by penicillins (19.1%), aminoglycosides (11.7%) and quinolones (11.1%) (Erbay et al. 2003). Another study showed that cephalosporins were the most frequently prescribed antibiotics (34%) followed by penicillins (33%), aminoglycosides (16%) and fluoroquinolones (6%) (Palikhe 2008). A study from Jordan revealed that penicillins (46%) and macrolides (39%) were the most frequently prescribed antibiotic classes (Al-Niemat et al. 2014). With regard to individual antibiotics, the findings of our study revealed that ceftriaxone (71.8%) contributed the highest percentage share amongst all the antibiotics followed by cefotaxime (5.6%), metronidazole (4.7%), amoxicillin (4.7%), ciprofloxacin (4.2%) and moxifloxacin (1.9%). An Indian study showed that the highest prescribed antibiotics were cefixime (37.98%), ceftriaxone (7.97%), azithromycin (6.33%) and gentamicin (6.25%) (Khan et al. 2011). In another study, azithromycin contributed the highest percentage share (97%) of the total antibiotics (Al-Niemat et al. 2014). It is clear from this body of literature that the extent of antibiotic prescription is high but that there is considerable variation in the number and types of antibiotics selected to prescribe. What is concerning from our study is the selection of very high powered cephalosporins first line. This study provides the platform to implement policy that should result in a change in this prescribing behavior. In this regard the study is very relevant and has implications for policy and practice that can make a difference in Pakistan.

The increasingly growing threat of AMR and the lack of a significant pipeline of new antibiotics in development has drawn attention toward multi-drug therapies (Golan et al. 2011). In the majority of cases infections are usually cleared within a few days of antibiotic treatment with a single agent, but severe and complicated infection may require longer treatment with a combination of multiple antibiotics (Nicolle and Committee 2005). We found that the most commonly prescribed antibiotic combinations were ciprofloxacin with metronidazole (52.1%) and ceftriaxone with metronidazole (38.8%). In the field of medicine, the multi-drug antibiotic therapies are usually sought to achieve broader antibacterial spectrum (Ejim et al. 2011). Different occasions where these combination therapies have been shown to be more effective include; synergism; prevention of AMR; and as empirical therapy for poly-microbial infections. The most commonly anticipated drug–drug interactions with antibiotic combinations are antagonism, addition and synergism (Rybak and McGrath 1996). In most cases, synergistic effects are considered for treatment failure cases or when the incidence of AMR development is most likely (Cremieux and Carbon 1992). But some recent studies have reported that antimicrobial synergistic combinations may speed up the process of AMR development (Pena-Miller et al. 2013).

There are certain other risks and adverse effects associated with the use of antibiotics and antimicrobial combinations. The major associated risks include development of super-infections, augmented toxicity and greater cost. The well described adverse effects include hypersensitivity reactions, diarrhea, nephrotoxicity and coagulopathy (Rybak and McGrath 1996). Published studies have emphasized the rational use of antibiotics (alone or in combination) to prevent the AMR development (McGowan 1983), improve quality of patient care (Shao-Kang et al. 1998) and to minimize the cost of therapy (Segade 2000).

## Conclusion and recommendations

The current study demonstrates irrational prescribing practices in the A & E department of a tertiary level hospital in Pakistan. The findings in terms of prescribing indicators are divergent with the expected norms. Cephalosporins contributed the highest percentage share amongst all the antibiotic classes, while ceftriaxone was the most commonly prescribed antibiotic. The extent of antimicrobial prescribing in this setting raises concerns of AMR and highlights an ignorance of doctors towards their prescribing behavior.

This study contributes to the international literature by addressing medicines use in the context of the A & E department; a point of first contact with the health system in many developing countries. Further, this study adds to the body of knowledge surrounding antibiotic use in this A & E setting and compares use in Pakistan with other developing countries. The study adds further evidence that policy-makers, hospital administrators, doctors, nurses and pharmacists should all be wholly concerned about the developing world’s ongoing contribution to AMR. There are few antibiotic medicines in the drug development pipeline and therefore few therapeutic solutions when current agents stop being effective. The strategy must immediately be focused on curbing the current rise in AMR.

The study findings support a recommendation that continuous training and educational programs for medical staff concerning rational use of injections and antibiotics should be implemented and monitored so that the required changes in prescribing become sustainable. A feedback monitoring system on doctors’ antibiotic prescriptions would be expected to significantly improve their prescribing behavior. Knowledge and compliance with updated clinical guidelines is also recommended to enhance the degree to which generic prescribing occurs.

Further studies are encouraged to find the reasons for irrational use of medicines and to demonstrate the factors driving the irrational prescribing of antimicrobials by the physicians at BHV. Appropriate interventions regarding rational prescribing of antimicrobials should be designed and implemented in the hospital and an evaluation is required to determine whether there has been any improvement in the prescribing practices of physicians.

## Limitations of the study

There are limitations with pharmaco-epidemiological approaches that compare studies across countries where the hospital context might be different. However, the authors are left with few options when the literature is scarce and limited within the context of A and E antibiotic prescribing in developing countries. In addition to international comparisons the findings from this study should not be generalized to the whole of Pakistan; but they do give a robust indication of what is happening in one hospital A and E department and may precipitate other hospitals in Pakistan to begin an audit which will give a sense of whether the high use of IV ceftriaxone is a national phenomenon. Based on the fact that a uniform healthcare policy is implemented throughout the country, and medical graduates from various institutions are working in Bahawalpur city the practices may be similar to A & E departments of other tertiary care hospitals in Pakistan. This needs to be further instigated and if it is the case then a national policy will need to be developed to address this issue. In this study, we did not explore the reasons for irrational drug use, which could be considered in future studies.

## Abbreviations

A & E: Accident and Emergency
ADR: adverse drug reaction
AMR: antimicrobial resistance
BVH: Bahawal Victoria Hospital
EDL: essential drugs list
INRUD: International Network for the Rational Use of Drugs
SPSS: Statistical Package for Social Sciences
WHO: World Health Organization
